# A53 A NOVEL PH-SENSITIVE OPIOID ANALOGUE PROVIDES SUSTAINED ANALGESIA DURING COLONIC INFLAMMATION AND LACKS COMMON SIDE EFFECTS

**DOI:** 10.1093/jcag/gwab049.052

**Published:** 2022-02-21

**Authors:** C E Degro, N N Jiménez-Vargas, Q K Tsang, Y Yu, C Stein, N W Bunnett, A E Lomax, D E Reed, S Vanner

**Affiliations:** 1 GIDRU, Queen’s University, Kingston, ON, Canada; 2 Medicine, Queen’s University, Kingston, ON, Canada; 3 Queen’s University, Kingston, ON, Canada; 4 GIDRU Wing, Kingston General Hospital, Kingston, ON, Canada; 5 Charite Universitatsmedizin Berlin, Berlin, Berlin, Germany; 6 New York University, New York, NY

## Abstract

**Background:**

Opioids provide effective pain relief during flares of inflammatory bowel disease but are limited by serious side effects. We showed that acute administration of a novel pH-sensitive opioid agonist, NFEPP, had potent analgesic effects in inflamed acidified colonic tissues without opioid typical side effects. However, the effects of repeated application of NFEPP during the course of an acute flare of colonic inflammation are unknown.

**Aims:**

To assess the analgesic and side effect profile resulting from repeated NFEPP applications during the course of acute colitis.

**Methods:**

Acute colitis in C57BL/6 mice was induced via 2.5% dextran sulfate sodium dissolved in drinking water for 5 days. Mice were then randomly group assigned to either vehicle, fentanyl or NFEPP. Drugs and vehicle were administered sc. BID for 5 days (0.4 mg/kg), with a final injection on Day 6 (Day 1–6). Visceral nociception was evaluated by performing visceromotor responses (VMRs) to colorectal distension at different time points during colitis. Side effects were assessed using 1) a combination of oral contrast-enhanced abdominal CT-scans (before/after treatment) and defecation assessments to analyze gastrointestinal transit and isometric tension recordings to evaluate colon motility 2) pulse oximeter measurements to reveal cardiorespiratory effects. Inflammation was assessed by histological scoring, myeloperoxidase activity assay and tissue pH measurement of colon samples.

**Results:**

NFEPP decreased VMRs to colorectal distension over the entire period of acute DSS colitis (Day 6: 40.54%, p<0.001, reduction compared to baseline). However, strongest VMR inhibition was observed at Day 3 (66.7%, p<0.05), concordant with the peak of inflammation (MPO: 3.5 U/mg tissue, p<0.001; tissue Damage-Score: 3.0, p<0.001; tissue pH: ΔpH= -0.28, p=0.001, all compared to healthy control). Treatment with NFEPP did not delay gastrointestinal transit (GIT, p=0.37, compared to baseline) nor fecal output (p=0.45, compared to vehicle) whereas fentanyl decreased transit (GIT: p<0.05; fecal output: p<0.05). Colonic contractile responses evoked by electrical field stimulation were reduced in fentanyl treated mice compared to NFEPP (10 Hz: p<0.05). Fentanyl significantly reduced oxygen saturation (15 min: 85% SpO2, p<0.01) and caused a sustained reduction in heart rate at Day 2 (60 min: Δ BPM -91.8 from baseline, p=0.001) whereas NFEPP did not affect oxygen saturation (p=0.95) and revealed minor, transient effects on heart rate (15 min: Δ BPM -75.6, p<0.01) which recovered after 60 min.

**Conclusions:**

Prolonged NFEPP administration effectively inhibits visceral nociception during acute colitis in a preclinical mouse model without altering gastrointestinal transit, colon motility and oxygen saturation.

**Funding Agencies:**

CCC

